# Oral nicotinamide provides robust, dose-dependent structural and metabolic neuroprotection of retinal ganglion cells in experimental glaucoma

**DOI:** 10.1186/s40478-024-01850-8

**Published:** 2024-08-23

**Authors:** Gloria Cimaglia, James R. Tribble, Marcela Votruba, Pete A. Williams, James E. Morgan

**Affiliations:** 1 School of Optometry and Vision Sciences, Cardiff University, Maindy Road, Cardiff, Wales UK; 2 Department of Clinical Neuroscience, Division of Eye and Vision, St. Erik Eye Hospital, Karolinska Institutet, Stockholm, Sweden; 3 University Hospital of Wales, Heath Park, Cardiff, Wales UK

**Keywords:** Dendrite, DiOlistics, Glaucoma, NAD, Metabolomics, Nicotinamide, Optic nerve, Retina, Retinal ganglion cell

## Abstract

A compromised capacity to maintain NAD pools is recognized as a key underlying pathophysiological feature of neurodegenerative diseases. NAD acts as a substrate in major cell functions including mitochondrial homeostasis, cell signalling, axonal transport, axon/Wallerian degeneration, and neuronal energy supply. Dendritic degeneration is an early marker of neuronal stress and precedes cell loss. However, little is known about dendritic structural preservation in pathologic environments and remodelling in mature neurons. Retinal ganglion cell dendritic atrophy is an early pathological feature in animal models of the disease and has been demonstrated in port-mortem human glaucoma samples. Here we report that a nicotinamide (a precursor to NAD through the NAD salvage pathway) enriched diet provides robust retinal ganglion cell dendritic protection and preserves dendritic structure in a rat model of experimental glaucoma. Metabolomic analysis of optic nerve samples from the same animals demonstrates that nicotinamide provides robust metabolic neuroprotection in glaucoma. Advances in our understanding of retinal ganglion cell metabolic profiles shed light on the energetic shift that triggers early neuronal changes in neurodegenerative diseases. As nicotinamide can improve visual function short term in existing glaucoma patients, we hypothesize that a portion of this visual recovery may be due to dendritic preservation in stressed, but not yet fully degenerated, retinal ganglion cells.

**Supplementary Information:**

The online version contains supplementary material available at 10.1186/s40478-024-01850-8.

## Introduction

The capacity to maintain NAD pools has emerged as a key factor in a range of age-related neurodegenerative conditions including Alzheimer’s disease, Parkinson’s disease, Huntington’s disease, glaucoma, and amyotrophic lateral sclerosis [1–6]. Although the clinical presentation varies, these conditions share common pathophysiological features including mitochondrial dysfunction and bioenergetic insufficiency [7–9]. At the neuronal level this is manifest as a prolonged phase of dendritic and synaptic degeneration that precedes neuronal death [10, 11]. Dendritic integrity is a key factor for neuronal health as it ensures signal integration which, in turn, maintains activity related transcription and retinal circuitry. Dendritic degeneration therefore provides a clear and quantifiable index of neuronal health.

Retinal ganglion cells are the output neurons of the retina and are a useful tool for the study of dendritic degeneration within the CNS. Retinal ganglion cells are selectively damaged in in glaucoma, a common neurodegenerative disease affecting an estimated 80 million patients worldwide [12]. It remains a leading cause of irreversible vision loss.

We have previously reported metabolic dysfunction and mitochondrial abnormalities occurring prior to neurodegeneration in glaucoma (in glaucoma patients and glaucoma animal models) [13, 14]. These studies identified that the capacity to maintain retinal NAD pools declines in an age-dependent manner and renders retinal ganglion cells susceptible to intraocular pressure-related stress (a risk factor for glaucoma), driving neurodegeneration [13]. Preventing NAD depletion by the administration of nicotinamide (NAM; the amide of vitamin B_3_, and an NAD precursor through the NAD salvage pathway) is robustly neuroprotective in animal models [13, 15, 16]. Supporting this, glaucoma patients have been shown to have systemically low levels of nicotinamide (in sera [17]) and low NAD in peripheral blood mononuclear cells [18]. Nicotinamide administration can improve visual function in existing glaucoma patients [19] and long term RCTs to evaluate the beneficial effects of oral nicotinamide (3 g/d) in for glaucoma are underway in Australia, Singapore, Sweden, and the United Kingdom (NCT05405868, NCT05275738). Since dendritic and synaptic reinnervation could be a useful substrate for visual recovery in glaucoma, it is important to determine whether nicotinamide treatment can provide dendritic neuroprotection in glaucoma and what doses might be required and whether this is suitable both as interventional and prophylactic strategies.

To address this, we compared effects of the administering daily high dose NAM (600 mg/kg/d in rat equivalent to 6.3 g/d in a 70 kg human) and low dose NAM (200 mg/kg/d in rat equivalent to 2.1 g/d in a 70 kg human) in a rat model of glaucoma, using cell death and dendritic degeneration as readouts of retinal ganglion cell protection.

We demonstrate that NAM is effective at high and low dose in preventing retinal ganglion cell dendritic degeneration when given prior to the onset of ocular hypertension. When NAM is given after the onset of ocular hypertension, a comparable level of protection is only seen with the higher NAM dose. These findings are relevant in considering the dose of NAM in the treatment of glaucoma and other neurodegenerative conditions.

## Materials and methods

### Animal strain and husbandry

Experiments were performed in compliance with the Home Office Animals (Scientific Procedures) Act 1986, UK and the ARVO Guidelines for the Use of Animals in Ophthalmic and Vision Research. Animals were housed in pairs in environmentally enriched cages with a 12 h/12 h light/dark cycle with food and water available ad libitum. Adult, male Brown Norway rats (BN/RijHsd, ENVIGO), approximately 14 weeks of age were handled by a single investigator (GC) for 2 weeks prior to induction of elevated IOP. Animal handling was critical in ensuring that the animals were calm during IOP measurements and which had a positive influence on reduced the noise in IOP measurements.

In preparation of for oral ingestion, nicotinamide (NAM; Apollo Scientific Ltd, UK) was dissolved in sweetened (commercial caster sugar) drinking water (200 mg/kg/day, based on a pre-determined average water consumption). Higher doses were achieved by combining treated drinking water and a custom diet (9380 ppm NAM RM3 P diet, SDS) [15], to achieve 600 mg/kg/day based on a pre-determined g/day consumption of chow. NAM enriched water was shielded from external light and changed every 3 days. The chow was replenished as required. All animals were trained by a single investigator (GC) with positive reinforcement (*e.g.* muesli, nuts, watermelon) and enrichment time to reduce stress during handling.

### Induction and monitoring of ocular hypertension

Ocular hypertension (HT) was induced as described by Tribble et al. [20]. Rats were anesthetized with 5% isoflurane (Piramal Healthcare UK Ltd)/2 L O_2_/min and maintained at 2.5% throughout the procedure. The contralateral eye (normotensive control; NT) was protected by Viscotears gel (Bausch & Lomb) while the ipsilateral eye (experimental ocular hypertensive; HT) received 0.4% Oxybuprocaine eye drops (Bausch & Lomb). Magnetic microbeads (Dynabeads M-450 Epoxy; Thermo Fisher Scientific) were prepared in 1 × Hank’s Balanced solution (HBSS -CaCl_2_, -MgCl_2_, -phenol red; Gibco) and 8–10 μL of bead solution (2.7 × 106 beads/µL; 240 mg/mL) slowly injected into the anterior chamber using a 100 µL NanoFil Hamilton syringe with a 33G tribevelled needle (World Precision Instruments). During the injection, the beads were directed to block the iridocorneal angle within the anterior chamber using a hand-held Neodymium-Boron magnet directed to the limbus and moved through 360 degrees. Once the needle was removed, chloramphenicol (0.5%) eye drops (Bausch & Lomb) were applied to minimize the risk of infection.

### NAM treatment schedule

For rats pre-treated with NAM (prophylactic), NAM was provided 2 weeks prior to the induction of HT and maintained throughout the duration of the experiments (4 weeks). For rats treated with NAM after the induction of HT, NAM (intervention), NAM was given 3 days after microbead injection and maintained throughout the duration of the experiments (4 weeks). Rats were trained with positive reinforcement to allow intraocular pressure (IOP) measurements without anaesthesia or restraint with a rebound tonometer (TonoLab). All IOP measurements were taken within 2 h in the morning by the same investigator (GC) For HT controls (no NAM treatment) and NAM intervention rats, IOP baseline measurements were taken 1 week preceding the induction of HT (recording every other day) and thereafter until the day prior to euthanasia. For prophylactic treatment, IOP baseline measurements were taken 1 week preceding the treatment and thereafter until the day before euthanasia. IOP recordings were performed between 7 and 9 am to limit the effect of the diurnal variation on IOP.

### Analysis of retinal ganglion cell dendritic structure

To analyse retinal ganglion cell (RGC) degeneration, rats were euthanized by increasing the concentration of ambient CO_2_ followed by cervical dislocation. Eyes were immediately enucleated, and the corneas marked with a hand-held cautery to maintain orientation during dissection. The eyes were then immediately transferred into ice-cold HBSS and the retinas rapidly dissected and placed, ganglion cell layer up onto a microscope slide for DiOlistic labelling [21, 22].

Retinal ganglion cell dendritic structure was assessed in flat-mounted retinas by Diolistic labelling of individual retinal ganglion cell dendritic tree (Helios gene gun system, Bio-rad). 80 mg of 1.7 μm M-25 Tungsten particles (Bio-rad) were coated with methylene chloride (400 μL)-dissolved 1,1’-Dioctadecyl-3,3,3′3’-Tetramethylindocarbocyanine Perchlorate (DiI, 2 mg; Invitrogen) and 3,3’- Dioctadecyloxacarbocyanine Perchlorate (DiO, 4 mg; Invitrogen) or 1,1'-Dioctadecyl-3,3,3',3'-Tetramethylindodicarbocyanine, 4-Chlorobenzenesulfonate Salt (DiD, 1 mg; Invitrogen). Coated particles were then distributed onto the interior surface of Tefzel tubing (30 cm; Bio-Rad) which was then cut into 12 mm segments. DiI/DiO- or DiI/DID-coated tungsten particles were delivered (120–130 psi, helium) with the gene gun to the retinal flat mount, 5 cm from the retina surface through a 3 μm polyethylene terephthalate membrane (Fisher Scientific) to prevent tungsten particle clumping. Flat mounted retinas were then incubated for 30 min in Neurobasal-A medium (Gibco; 37° C, 5% CO_2_), fixed for 1 h in 4% paraformaldehyde (PFA), washed in 1 M PBS, nuclei labelled with Hoescht (1:1000 in PBS) and washed again. Retinas were then mounted with FluoroSave (Millipore) mounting media, coverslipped, sealed with nail polish, and dried at room temperature for 1 h.

Images of retinal ganglion cell dendritic fields were taken with a Zeiss LSM 780 confocal microscope (Carl Zeiss; 20X magnification, 0.345 μm/pixel, 1 μm *z*-thickness). RGCs were identified by their location in the ganglion cell layer, the presence of an axon projecting to the optic nerve, and a dendritic tree extending within the inner plexiform layer (IPL). Only the cells that fulfilled this selection criteria were imaged and traced. Complete dendritic fields were reconstructed using Imaris software (version 9.3.1, Bitplane), where individual retinal ganglion cell dendritic fields were manually selected as areas of interest and dendrites automatically traced using the filament tool. Any tracing errors were corrected manually. The dendritic field area was calculated by computing the convex hull of the bounded by the dendrite tips. Statistical parameters were exported from Imaris for Sholl analysis at set intervals of 10 μm.

All experimental procedures by the same investigator (GC). To avoid any bias, the treatment condition was masked during dendritic reconstruction and unmasked following analysis of the raw data. It is important to note that we analysed all retinal ganglion cells as a single group and not by class. The use of DiOlistics precludes the precise subtyping of retinal ganglion cell subtypes which can be problematic in retinal models of disease. We adopted the approach as used by others to present data on a larger number of cells *e.g.* [23] to analyse dendritic change in a glaucoma model. We have demonstrated previously that DiOlistic labelling does not show any bias for labelling by class [24].

### Nuclear counts

Six selected regions (3 superior, 3 inferior; 2048 × 2048 pixel, pixel size 0.345 µm/pixel) from a tiled image of each retina were analysed on Imaris. Ganglion cell layer nuclei were identified automatically using the spot tool (5.5 μm diameter) and the density normalized to nuclei/mm^2^.

### Low molecular weight enriched metabolomics

Metabolomics were performed as previously described [15]. Briefly, rats were euthanized by inspiration of an increasing concentration of CO_2_ followed by cervical dislocation. Brains were removed and optic nerves rapidly dissected and separated at the chiasm in NT (right eye) and HT (left eye) immediately after death. The samples were then weighed and snap frozen in liquid nitrogen. The tissue was stored at – 80 °C and shipped on dry ice to the Swedish Metabolomics Centre (Umeå, Sweden) for processing. Prior to analysis, 200 µL of 90:10 MeOH:H_2_O was added to each frozen sample on dry ice, acid washed glass-beads (425–600 μm, Sigma Aldrich) were added to constitute 50% v/v of the MeOH:H_2_O solution and samples were disrupted by shaking at 30 Hz for 2 min (Mixer Mill MM400) using pre-chilled holding blocks (4 °C). Following cell disruption samples were centrifuged at 4 °C using 14.000 RPM for 15 s. To obtain a 60:40 ratio of MeOH:H_2_O, 100 µl of H2O was added to the samples followed by shaking at 30 Hz for 15 s (Mixer Mill MM400) using pre-chilled holding blocks. Samples were then centrifuged at 4 °C at 14,000 RPM and the supernatant transferred into LC–MS vials (Thermo Fisher) and analysed immediately. 10 µL was injected into an Agilent 1290 UPLC-system connected to an Agilent 6546 Q-TOF mass spectrometer with an Agilent Jet Stream electrospray ionization (ESI) source. Data were collected in negative ionization mode with ESI settings: Gas temperature 150 °C, drying gas flow 16 L/min, nebulizer pressure 35 psi, sheet gas temperature 350 °C, sheet gas flow 11, Vcap 4000, nozzle voltage 300 V, Fragmentor 380, Skimmer1 45 V and OctapoleRFPeak 750 V. Metabolites were separated using a HILIC HPLC column (iHILIC-Fusion(+), 100 × 2.1 mm, 3.5 µM, 100 Å, Hilicon AB). HILIC elution solvents were (A) H2O, 50 mM ammonium formate and (B) 90:10 Acetonitrile:[50 mM ammonium formate in H2O]. Chromatographic separation was achieved using the following linear gradient (flow rate 0.4 mL/min), min 0: 90% B; min 4: 85% B, min 5: 70% B, min 7: 55% B, min 10: 20% B, min 10.01: 90% B, min 15: 90%. Fifty four low molecular weight metabolites that could be verified with standards were detected. Metabolites were quantified as area under the curve of the mass spectrometry peak. Although internal standard data for AMP showed a stable intensity suggesting that normalization was not necessary, samples were normalized to tissue weight. Data were analysed using MetaboAnalyst [25, 26]. Groups were compared by two-sample *t*-test with an adjusted *p* value cutoff of 0.05 considered significant. Principal component analysis was undertaken using Pareto scaling. Pathway analysis was performed in MetaboAnalyst using the *Rattus norvegicus* KEGG library.

### Statistical analysis

Statistical analyses were performed in GraphPad Prism (version 9.4.1). For boxplots: whiskers indicate minimum and maximum values; box plots represent 1st quartile, median, and 3rd quartile; dots indicate individual values. The normality of data sets was tested using the *Shapiro–Wilk* test. Two group comparisons were evaluated by *t*-test or *Mann–Whitney U* test as appropriate. To compare three or more groups one-way *ANOVA*, two-way *ANOVA*. or *Kruskal–Wallis* one-way *ANOVA* were used as appropriate, followed by *Tukey post-hoc* or *Dunn’s* test respectively. Significance was denoted as follows: ns = *p* > 0.05; *p < 0.05; **p < 0.01; ***p < 0.001; ****p < 0.0001. Power calculations were performed on G*Power (version 3.1). A priori power calculations were performed to decide the minimum sample size; post hoc power calculations were performed to verify the goodness of the data. Samples sizes are shown in Table 1.

**Table 1:** Sample sizes for all experiments

Group abbreviation	Group descriptor	Animal number	Retina number	Total number of RGCs	Mean RGC number/retina ± SD
C.NT	Control normotensive	11	22	182	16.81 ± 2.77
NT.LNAM	Normotensive low dose NAM	6	12	115	19.16 ± 3.81
NT.HNAM	Normotensive high dose NAM	6	12	113	18.83 ± 2.74
G.HT	Glaucoma injected control	11*	11	83	9.45 ± 3.60
N.HT	Glaucoma uninjected control	11*	11	66	7.72 ± 2.80
Proph.G.HT.LNAM	Prophylactic low dose NAM glaucoma injected	6*	6	54	10 ± 2.87
Proph.N.HT.LNAM	Prophylactic low dose NAM glaucoma uninjected	6*	6	40	6.66 ± 2.56
Proph.G.HT.HNAM	Prophylactic high dose NAM glaucoma injected	12*	12	136	13 ± 3.41
Proph.N.HT.HNAM	Prophylactic high dose NAM glaucoma uninjected	12*	12	102	9.1 ± 3.76
Int.G.HT.LNAM	Intervention low dose NAM glaucoma injected	6*	6	79	13 ± 4.6
Int.N.HT.LNAM	Intervention low dose NAM glaucoma uninjected	6*	6	62	11.14 ± 2.82
Int.G.HT.HNAM	Intervention high dose NAM glaucoma injected	12*	12	104	11.07 ± 2.12
Int.N.HT.HNAM	Intervention high dose NAM glaucoma uninjected	12*	12	107	10.41 ± 3.43

*Injected and uninjected retinas were taken from the same animal

## Results

### The effect of NAM on uninjured retinal ganglion cells

**Fig. 1:**
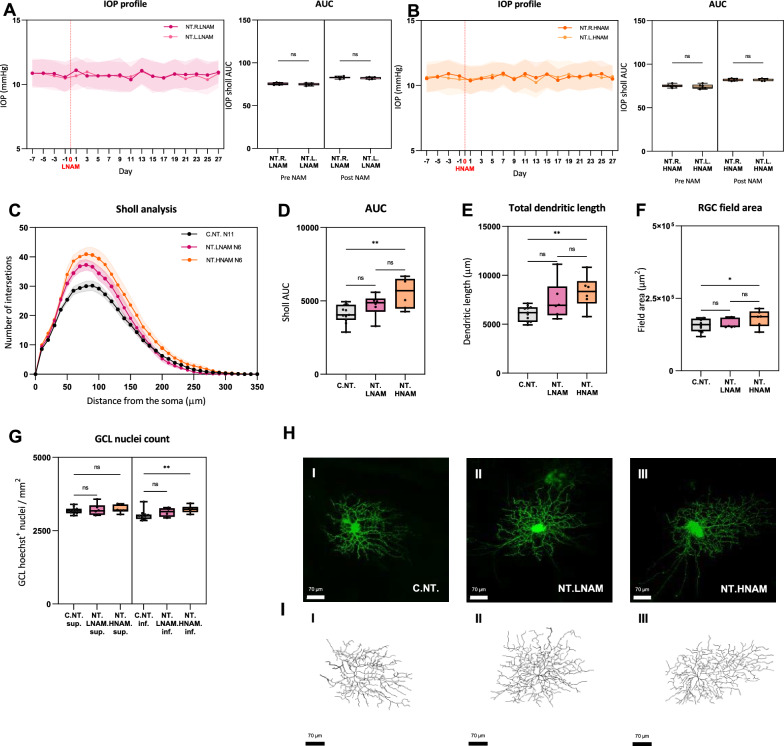
The effect of high and low dose NAM on uninjured retinal ganglion cells. **A**, **B** plots of intraocular pressure measured for 28 days prior to euthanasia. The IOP was not elevated in either eye. AUC: area under the IOP curve. **C** Sholl analysis showing the dendritic complexity for RGCs from untreated rats (C.NT), RGCs from rats treated with low dose NAM (NT.LNAM) or high dose NAM (NT.HNAM). Mean Sholl profiles are higher for the H.NAM group. *p* > 0.05; *p < 0.05; **p < 0.01. **D** The Area under the Sholl curve for plot C. **E**, **F** show the total dendritic area and RGC field area changes. **G** Mean cell (nuclear stain, the retinal ganglion cell layer) counts from 3 regions in the superior and inferior retina for the 3 groups confirming no change in counts. **H** Representative DiOlistically labelled cells from controls, low and high NAM groups with corresponding wireframe dendritic reconstructions. Scale: 70 μm. For all plots: *p* > 0.05; *p < 0.05; **p < 0.01. The number of cells, animals and retinas in each group is summarised in Table 1

We first assessed the effect of nicotinamide (NAM) treatment on the dendritic complexity of uninjured retinal ganglion cells (RGCs). Rats were treated with either high dose (600 mg/kg/d; HNAM) or low dose (200 mg/kg/d; LNAM) nicotinamide in food and water for 4 weeks. The data are summarised in Fig. 1. Sholl analysis indicated a tendency for greater dendritic area and neurite length following high and low dose NAM (Fig. 1C–F) without any changes in the number of RGCs (Fig. 1G). Oral NAM had no effect on intraocular pressure as measured every 3 days for 4 weeks following NAM administration.

### Elevated intraocular pressure drives dendritic atrophy and RGC loss

**Fig. 2:**
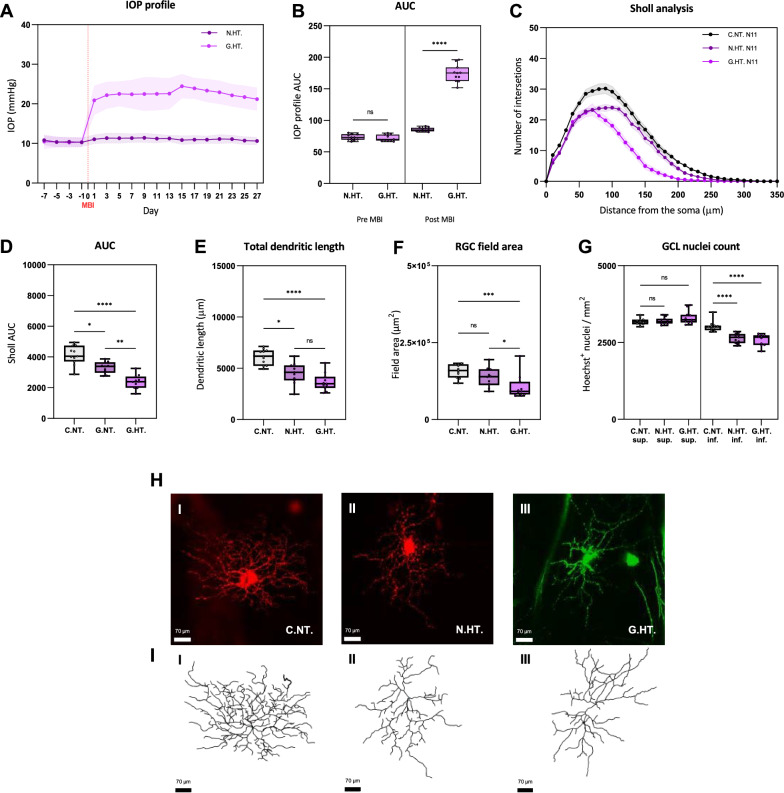
Unilateral IOP elevation induced dendritic atrophy in the treated and fellow eye. **A** Change in IOP following the induction of unilateral ocular hypertension following a single injection of microbeads. Each IOP value is the mean of 3 measurements. The plots show the mean IOP change for rats with elevate IOP in one eye (G.HT) compared with the normotensive fellow eye (N.HT). Shaded areas ± 1 SEM. MBI (red): microbead injection. **B** The areas under the IOP curve (AUC) confirms the substantial increase in IOP following MBI. **C** Sholl plots for labelled RGC from the eyes with elevated IOP (G.HT), and the normotensive controls from the same animals ((N.HT). For comparison we have also plotted the Sholl profile for RGCs from a naive rat (data plotted in Fig. 1). Grey shaded area: SEM. **D** Summary AUC for the Sholl plots demonstrating significant reductions in AUC for the hypertensive and fellow normotensive eye compared to naive controls. **E**, **F** Summary measurements of the extent of the dendritic tree showing reduction compared to naive controls. **G** The RGC nucleus count was reduced in both the hypertensive and fellow eye, for superior and inferior retina. **H, I** Representative DiOlistically labelled cells from controls, low and high NAM groups with corresponding wireframe dendritic reconstructions. Scale: 70 μm. *ns* = *p* > 0.05, * = *p* < 0.05, ** = *p* < 0.01, *** *p* < 0.001, **** *p* < 0.0001

Since dendritic atrophy and RGC loss are the principal readouts for the effect of NAM we next confirmed that our model of experimental glaucoma caused RGC damage, affecting these parameters following a moderate and sustained increase in IOP. Figure 2A shows the mean IOP profile following a single injection for the treated (G.HT) and fellow non-treated (N.HT) eye. Moderate IOP elevation was achieved in all animals with minimal fluctuation. The change in RGC complexity after 28 days is in shown in Fig. 2C. The greatest reduction in dendritic complexity, affecting mostly higher order dendrites was seen in the eyes with ocular hypertension (G.HT). However, we also observed a modest reduction in dendritic complexity for the fellow (normotensive) eye in these animals (N.HT). This observation is consistent with our earlier report of microglial activation in the contralateral normotensive eye, confirming that that while the contralateral eye can be used as one form of control, RGCs from a naive rat provide a better reference control [27].

### Starting nicotinamide treatment before the induction of ocular hypertension provides robust retinal ganglion cell protection at both high and low doses

**Fig. 3:**
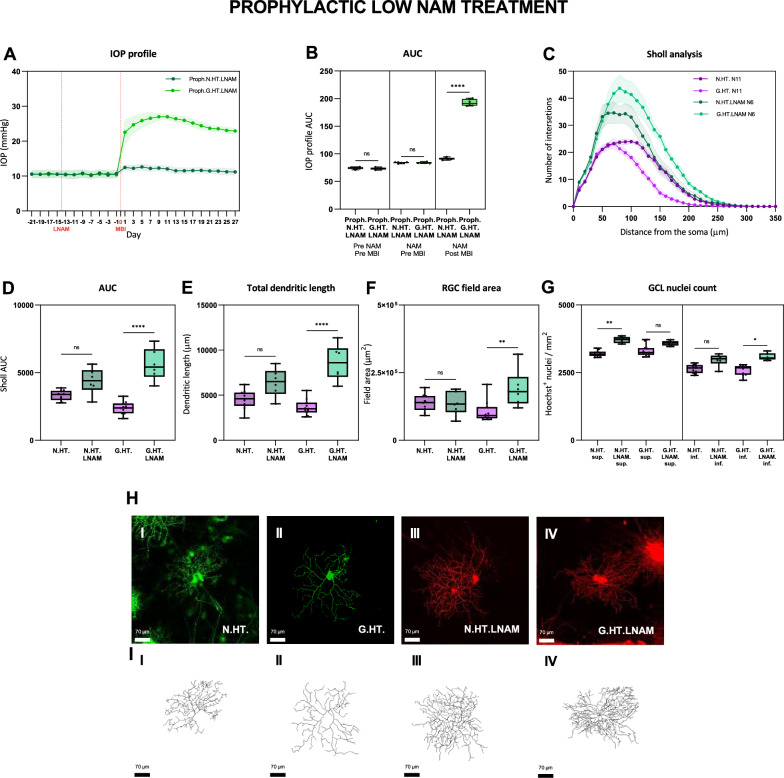
Retinal ganglion cell dendrite changes following prophylactic low dose NAM (LNAM) treatment compared to untreated unilateral (N.H) glaucoma. The IOP profiles and Sholl analysis follow the conventions for Fig. 2. H. Typical labelled cells with wireframe reconstructions. Scale: 70 μm. *ns* = *p* > 0.05, * = *p* < 0.05, ** = *p* < 0.01, *** = *p* < 0.001, **** = *p* < 0.0001

We next assessed the effect of treating rats with NAM prior to the induction of ocular hypertension (‘Prophylactic NAM’). Figure 3 shows the effect of ocular hypertension on RGCs in rats given low dose NAM 2 weeks prior to the induction of HT (‘LNAM’). Since we observed that unilateral ocular hypertension induced dendritic atrophy and cell loss in the hypertensive and fellow eyes, we have included data from naive (uninjected) animals as a baseline. Figure 3A confirms the low variation in IOP measurements following the induction of ocular hypertension for both the control and injected eyes. We include 3 weeks of IOP measurements to cover the period preceding NAM treatment. The increase in IOP and IOP AUC was significant and sustained in all animals (Fig. 3B). The Sholl plots in Fig. 3C demonstrate that dendritic complexity was greater in RGCs from eyes with high intraocular pressures (G.HT.LNAM) compared with the fellow normotensive eyes (N.HT.LNAM). Both were significantly greater than either the hypertensive or normotensive eyes (G.HT and N. HT respectively) from untreated (no NAM given) animals as plotted in Fig. 2 and shown here for comparison. Figure 3E–G show additional Sholl summary data confirming the statistical significance of these findings.

Cell counts in the GCL layer show that these were higher for both the superior and inferior retina compared with untreated ocular hypertensive eyes. The eyes showed greater GCL cell preservation following LNAM treatment compared with untreated animals (Fig. 3G). We also observed a slightly higher GCL count in the fellow eyes relative to controls following NAM treatment. It is useful to note that treatment with NAM did not affect the IOP profile, indicating that the protective effect occurred in the presence of elevated IOP. Figure 3H and I shows typical labelled cells. The degree of dendritic degeneration is striking as is the complexity of the dendritic trees in the NAM treated animals from both the high and normal pressure eyes.

**Fig. 4:**
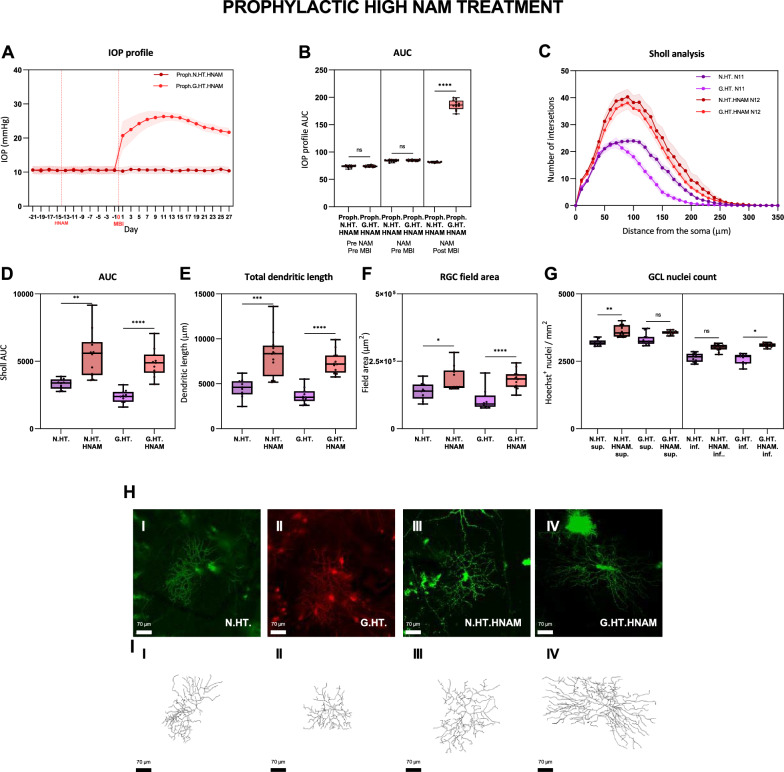
Retinal ganglion cell dendrite changes following prophylactic high dose NAM (HNAM) treatment compared to untreated unilateral (N.H) glaucoma. The IOP profiles and Sholl analysis follow the conventions for Fig. 2. H. Typical labelled cells with wireframe reconstructions. Scale: 70 μm. *ns* *p* > 0.05, * = *p* < 0.05, ** = *p* < 0.01, *** = *p* < 0.001, **** = *p* < 0.0001

We next determined whether the effect of pretreatment was dose related. While there is likely to be a ceiling to which RGC dendritic complexity can be enhanced, it is notable that the protective effect on RGCs from the fellow normotensive eye was partial. We therefore repeated the experiments described in Fig. 3 with high dose NAM (`HNAM’; Fig. 4). We again observed a substantial protective effect. While the protective effect on RGCs from the hypertensive eye was similar to that seen from the low dose NAM, the RGCs from the normotensive fellow eye now overlap with those from the hypertensive eyes. These observations suggest that for pretreatment with NAM the protective effects are dose related. A sub-analysis of RGC types into ON and OFF centre RGCs on the basis of arborisation in the IPL demonstrated comparable protective effects with a trend for this to be greater in OFF-centre RGCs (not statistically significant; data not shown).

Regression analysis to explore interactions between Sholl AUC and IOP indicated that the IOP profile had a significant negative effect on the area under the Sholl curve, confirming IOP alone a predictor of dendritic atrophy. We did not find any significant influence of NAM on IOP; NAM does not therefore appear to exert its effects through any modulation of IOP.

### Interventional NAM treatment confers RGC protection at high but not low dose

**Fig. 5:**
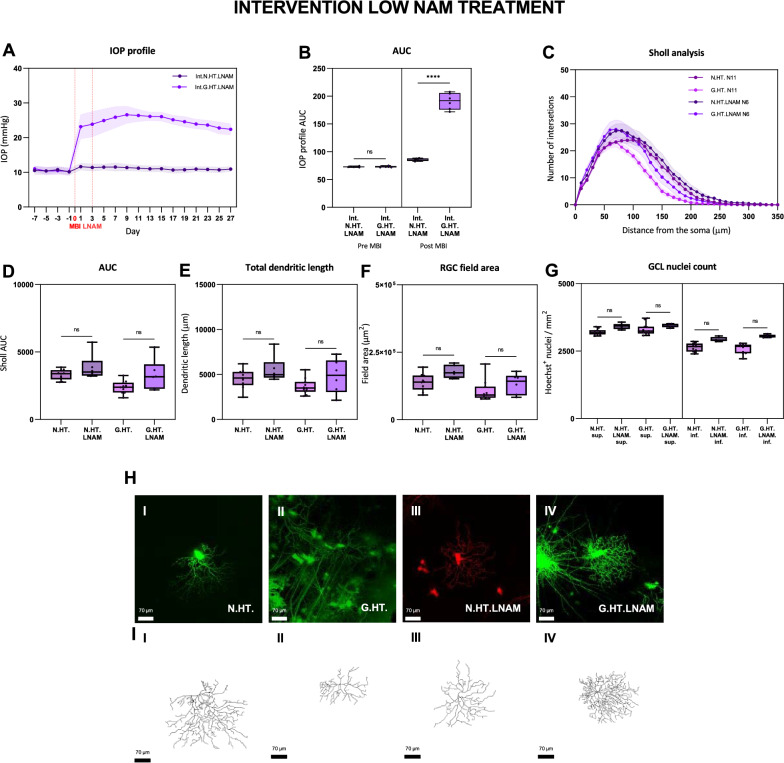
Retinal ganglion cell dendrite changes following induction of unilateral ocular hypertension with interventional low dose NAM treatment compared to untreated unilateral glaucoma. The IOP profiles and Sholl analysis follow the conventions for Fig. 2. The red lines on the IOP plot (A) indicate the start of NAM treatment 3 days after the induction of elevated IOP

While pretreatment provides a useful measure of the potential for NAM to protect RGCs, it does not reflect clinical reality in that glaucoma patients are treated following a diagnosis or glaucoma or ocular hypertension. We therefore determined whether NAM given following the induction of OHT was protective. Figure 5 demonstrates the effect of oral NAM given daily at low dose 3 days after the induction of OHT (selected since this time point corresponds, in most cases to peak IOP). By contrast with pretreatment (prophylactic) NAM treatment we did not observe a significant protective effect on RGC dendrites (Fig. 5C). Analysis of GCL nuclear counts showed a trend for increase GCL counts with LNAM treatment though this did not reach statistical significance (Fig. 5G).

**Fig. 6:**
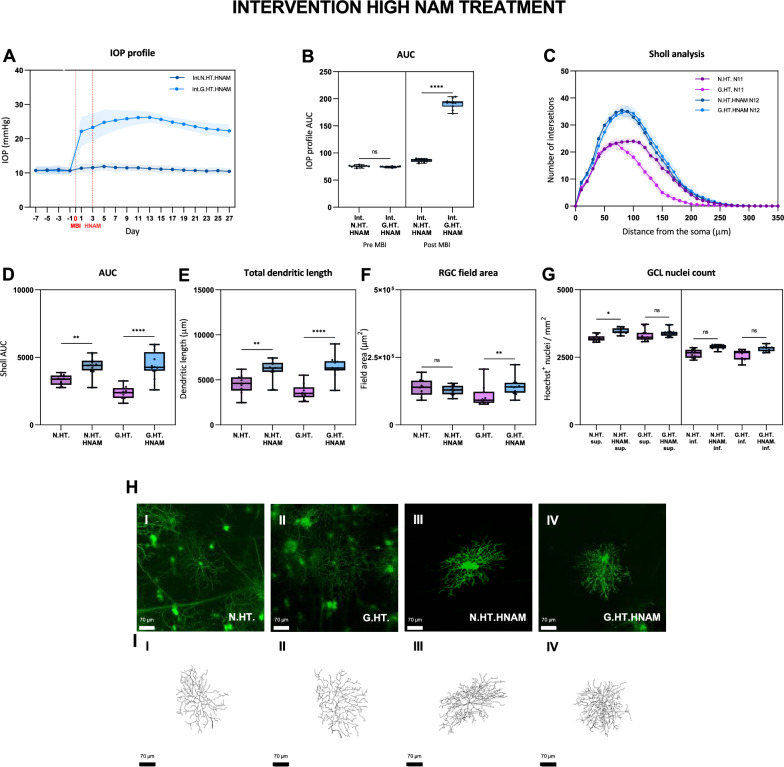
Retinal ganglion cell dendrite changes following induction of unilateral ocular hypertension with interventional high dose NAM treatment compared to untreated unilateral glaucoma. The IOP profiles and Sholl analysis follow the conventions for Fig. 2. The red lines on the IOP plot (A) indicate the start of NAM treatment 3 days after the induction of elevated IOP. *ns* = *p* > 0.05, * = *p *< 0.05, ** = *p*< 0.01, *** = *p *< 0.001, **** = *p *< 0.0001

We then assessed whether high dose NAM (HNAM) given 3 days following the induction of ocular hypertension was protective (Fig. 6). Figure 6C shows substantial protection of RGC dendrites and preservation of GCL nuclear counts (Fig. 6G) confirming that NAM mediated protection is dose related. We observed a similar protective effect for RGCs subdivided into ON and OFF classes (data not shown).

### Oral NAM exerts protective effects at the level of the optic nerve

Although we could evidence that the rats consumed the NAM, based on their daily intake and the absence of residual food within a 24 h period, it is important to confirm that sufficient NAM was delivered to the retina and optic nerve. Since the retinas were not available for further analysis following DiOlistics, we undertook a metabolomic analysis of optic nerve tissue from the eyes/retinas used for DiOlistics. We followed our low molecular weight metabolomics protocol [15] using the optic nerve as a surrogate for the RGC changes; the approach is reasonable because the bulk of the optic nerve comprises RGC axons. We used the high dose cohorts for analysis, given as an intervention or prophylactically, as above.

**Fig. 7:**
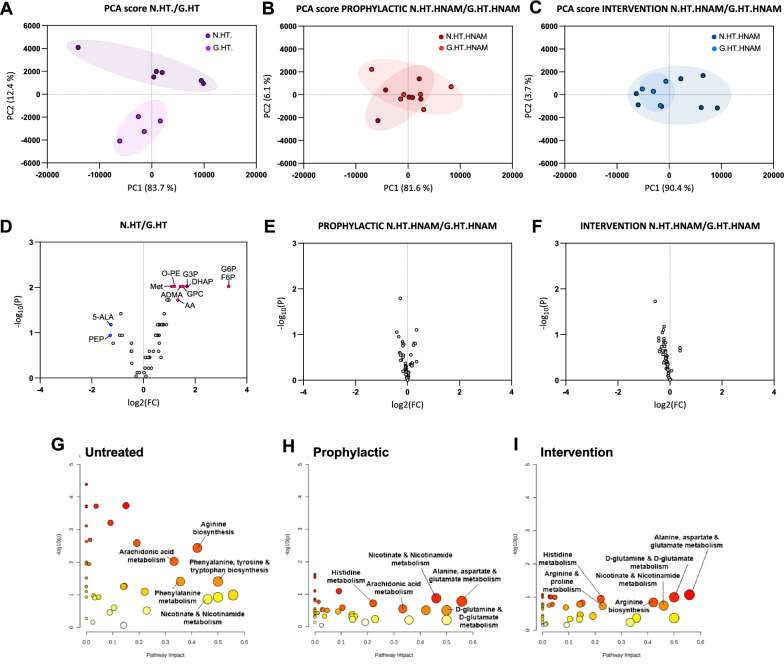
Metabolomic profiles of optic nerves following high dose NAM given prophylactically (before the onset of HT) or as an intervention (after the onset of HT). In each case the normotensive eye (N.HT) is compared with the hypertensive eye (G.HT) from the same rat. These plots demonstrate that the metabolic profiles for high pressure eye converge on the those for nomotensive eyes following high dose NAM treatment. **A**–**C** Principal component analysis of metabolomic profiles comparing the normal and hypertensive eyes from rats with induced ocular hypertension. **A** No NAM treatment. **B** Prophylactic High NAM treatment. **C** Interventional high NAM treatment. **D**–**F** corresponding Volcano plots of NT vs. HT (no NAM treatment) (**D**), NT vs. HT (prophylactic NAM treatment) (**E**), and NT vs. HT (interventional NAM treatment) (**F**) indicating the convergence of the metabolomic profile following NAM treatment (**E**, **F**) compared with no NAM treatment (**D**). No significant change in metabolite profile in HT eye was seen following NAM treatment when given at a prophylactic or interventional high dose. **G**–**I** Pathway analysis (MetaboAnalyst) was performed on detected metabolites and annotated using the Rattus norvegicus KEGG library (**G**) Pathway analysis of NT vs. HT (no NAM treatment; Untreated). **H** NT vs. HT (prophylactic NAM treatment; Prophylactic). **I** NT vs. HT (interventional NAM treatment; Intervention). Significant fold change (^>^2) are shown (red markers) for 5-ALA: 5-aminolevulinic acid; AA: arachidonic acid; ADMA: asymmetric dimethylarginine; DHAP: dihydroxyacetone phosphate F6P: Fructose-6-phosphate; G3P: glyceraldehyde-3-phosphate; GPC: glycerophosphocholine; G6P: glucose-6-phosphate; Met: methionine; O-PE: O- phosphoethanolamine; PEP: phosphoenolpyruvate. No significant fold changes are seen following prophylactic or interventional NAM at high dose (and none of the corresponding pathways are significantly enriched in these comparisons; **H** and **I**)

In untreated glaucomatous rats, principal component analysis (PCA) discriminated hypertensive eyes (HT) and contralateral normotensive (NT) control eyes as two distinct, non-overlapping groups (Fig. 7A). By contrast, the two NAM treated groups could not be discriminated from contralateral controls demonstrating a clear overlap of metabolic profiles consistent with robust metabolic protection (Fig. 7B and C). Ocular hypertension induced a clear metabolic change with 8 upregulated metabolites glucose-6-phosphate, glyceraldehyde-3-phosphate, arachidonic acid, glycerophosphocholine, methionine) and 2 significantly downregulated (phosphoenolpyruvate, 5-aminolevulinic acid) (Fig. 7D, Supplementary Material 1) resulting in significant enrichment of  12 pathways including arginine biosynthesis, fructose metabolism, pentose phosphate pathway, and glycolysis (Fig. 7G, Supplementary Material 2). There we no significant differences in metabolite profiles between NAM treatment group and OHT controls (Fig. 7E, F) confirming robust metabolites normalisation following NAM treatment. These changes were reflected in the pathway analysis (there are no significantly enriched pathways in the 'Prophylactic' or 'Intervention' contrasts shown in Fig. 7H and I).

**Fig. 8:**
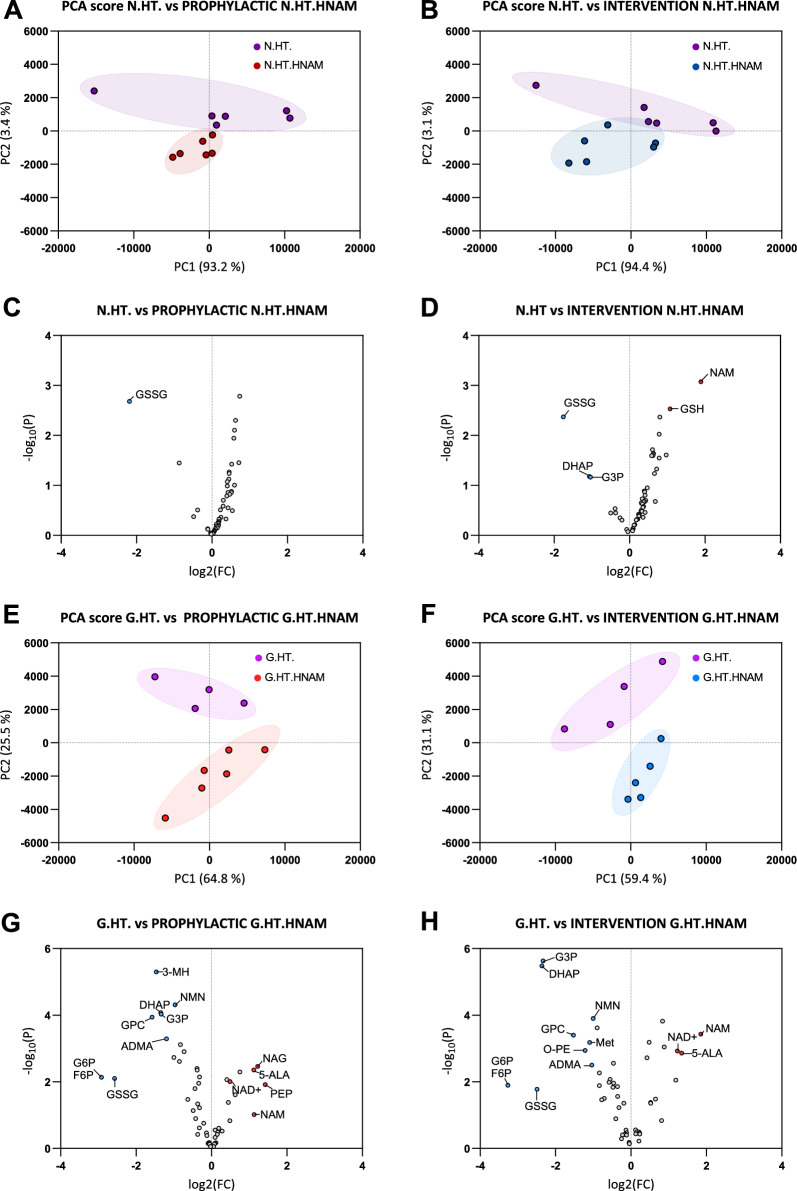
Metabolomic profiles of optic nerves following high dose NAM given prophylactically (before the onset of HT) or as an intervention (after the onset of HT). These plots demonstrate that the metabolic profiles for high pressure eyes treated with high dose NAM differ significantly from high pressure eyes not treated with high dose NAM. **A** and **B** Principal component analysis of metabolomic profiles from normotensive eyes (N.HT, high pressure in the other eye), not receiving NAM treatment, compared with normotensive eyes (N.HT, high pressure in the other eye) receiving prophylactic or interventional, high dose NAM) The corresponding volcano plots are shown (**C** and **D**). **E** and **F** Principal component analysis of metabolomic profiles from eyes with high pressure (G.HT, normal pressure in the other eye), not receiving NAM treatment, compared with hypertensive eyes (G.HT, normal pressure in the other eye) receiving prophylactic or interventional, high dose NAM) The corresponding volcan plots are shown in **G** and **H**

To determine the metabolic changes that occur in the RGC / optic nerve following NAM treatment we performed a subset analysis comparing control normotensive (NT; unoperated) contralateral eyes with and without NAM treatment (Fig. 8A–D) and injected OHT eyes with and without NAM treatment (Fig. 8E–H). We determined good discrimination based on PCA between all groups, again supporting a positive modulation of metabolism with NAM treatment. In control normotensive (NT; unoperated) contralateral eyes there was a clear shift in metabolic profiles based on the PCA for both prophylactic (Fig. 8A) and interventional (Fig. 8B) NAM treatment. Metabolic changes were most pronounced following prophylactic *vs* interventional treatment (Fig. 8C, D) dominated by increased NAM (as expected) and a shift in the GSH:GSSG ratio (low GSSG and high GSH following treatment). We next compared injected HT eyes with and without NAM treatment (Fig. 8E–H). We observed the same shift in GSH:GSSG ratio, a downregulation of G6P and F6P production, and a shift in the NAD salvage pathway following NAM treatment both as an interventional treatment (Fig. 8E, G) and as a prophylactic treatment (Fig. 8F, H).

The maintenance of a NAD pool is critical to neuronal health and survival. RGCs are highly dependent on the NAD salvage pathway to generate NAD from NAM through a two-step reaction (NAM to NMN by the enzyme NAMPT, and NMN to NAD by the enzymes NMNAT1 and NMNAT2) [28]. NAM did not change following the induction of HT, and oral NAM rapidly increased NAM levels in the optic nerve. This effect is most pronounced in the interventional treatment (Fig. 8A). Following 3 days of HT NMN rapidly rises in the optic nerve (Fig. 8B) with a concomitant decrease in NAD + (but not NADH) levels (Fig. 8C, D). This is also reflected in the ratio of NMN:NAD consistent with a pro-neurodegenerative phenotype (Fig. 8E). These effects are strongly reversed and normalized by both interventional and prophylactic NAM treatment (Fig. 8C–E). Collectively, these data demonstrate that NAM treatment provides robust metabolic- and neuro- protection for RGCs under ocular hypertension-induced stress.

## Discussion

Dendritic survival is essential for the preservation of neuronal function and the maintenance of neuronal circuitry [11, 29, 30]. Dendritic atrophy is one of the earliest events in the neurodegenerative cascade and is a characteristic pathological event in retinal ganglion cells in glaucoma [14, 30, 31]. Previous studies have indicated that this may be due to bioenergetic imbalance possibly due to mitochondrial dysfunction [13, 15] raising the possibility that the remediation of any associated NAD insufficiency could prevent retinal ganglion cell dendritic atrophy. Here we report that replenishing the NAD pool through oral nicotinamide (an NAD precursor through the NAD salvage pathway) treatment can provide robust, dose related dendritic protection in a rat model of experimental glaucoma.

In these experiments we used two experimental paradigms; prophylactic treatment (which we have previously shown to be neuroprotective in this model [15]) and a more clinically relevant interventional treatment (where treatment was started when intraocular pressure was at its peak; 3 days post-surgery). Two doses of nicotinamide were assessed with each paradigm; high dose nicotinamide (600 mg/kg/d in rat equivalent to 6.3 g/d in a 70 kg human) and low dose nicotinamide (200 mg/kg/d in rat equivalent to 2.1 g/d in a 70 kg human). Both doses provide robust neuroprotection for retinal ganglion cell dendritic fields when provided as a prophylactic treatment (*i.e.* providing a pro-neuroprotective environment prior to injury) whereas only the higher dose provided similar neuroprotection when given as an intervention. Collectively, these data confirm that dendritic atrophy occurs early in glaucoma and that it can be robustly prevented, or even reversed, by nicotinamide treatment. As dendrites are plastic (whereas the optic nerve cannot regenerate under normal physiological conditions), dendritic remodelling by NAM may be a potential avenue to vision maintenance or recovery in, at least, early glaucoma patients.

Current Phase III nicotinamide trials (NCT05405868 and NCT05275738) use a treatment dose of 3 g/d based on the lowest effective dose demonstrated in the DBA/2 J mouse model of glaucoma [13, 16]. The 3 g/d dose has been shown to be effective in increasing visual function in patients with established glaucoma [19]. It remains to be seen if this dose will be optimal for long-term retinal ganglion cell protection in these clinical trials. Since trials are enrolling new, mild, and moderate glaucoma patients, a subsequent meta-analysis will be useful in determining the efficacy of NAM treatment as a function of disease stage.

It is important to note that the CNS is typically non-regenerative and there are no current treatments that can fully restore damaged axon tracts all the way to terminal targets in the brain. However, retinal ganglion cell dendrites are plastic, allowing for the reintegration of neuronal circuits (*e.g.* via BDNF or insulin treatment [4, 32, 33]), and as such, dendritic recovery may provide a useful substrate for visual recovery in glaucoma. We previously identified in areas of the retina with mild visual field loss, dendritic atrophy in midget retinal ganglion cells, supporting the connection between dendritic atrophy on visual function and the potential for dendritic recovery as a driver for the recovery of vision in glaucoma.

During glaucoma retinal ganglion cells can be considered to be in a heterogeneous dysfunctional state; with a mixture of healthy, stressed, dysfunctional, dying, and fully degenerated retinal ganglion cells across the retina [34]. Previous clinical trials have demonstrated a transient increase in visual function in glaucoma patients following nicotinamide treatment (over a 12 week period) [19], and we hypothesize that part of this recovery may be by restoring bioenergetic balance and increasing dendritic plasticity / recovery in the inner retina. An interesting question with the protection of retinal ganglion cell structure is whether dendritic preservation or regeneration occurs (or potentially a mix of both given the heterogeneity in degenerative stages across individual retinal ganglion cells). Since some dendritic atrophy is likely to have occurred in the intervention group (NAM given 3 days post OHT) it is possible that we are seeing some dendritic regeneration. However, a more robust test of this possibility would be to determine the level of dendritic normalisation occurring following NAM treatment given after 4 weeks of elevated intraocular pressure—at a time point when substantial retinal ganglion cell degeneration has occurred. These are potential future experiments to determine whether NAM would be beneficial in late-stage glaucoma patients to maintain or enhance residual vision.

In the context of restoring the retinal ganglion cell energy balance, the critical role played by AMPK in the monitoring of cellular energy levels is of some relevance since AMPK activation (driven by lowered AMP levels) appears to be a factor driving RGC degeneration in a mouse model of glaucoma to the extent that AMPK inhibition is protective for RGC dendritic structure in experimental glaucoma [23]. Since AMPK activation also reduces mTORC1 activity (itself a driver of dendritic atrophy) it suggests that the rectification of energetic defects can be used synergistically to recover neuronal structure in glaucoma [35]. There is considerable interest in the use of insulin to recover mTORC function [32] and it is reasonable to consider that the coadministration of NAM would be reasonable in this model of treatment. Recent data supports the hypothesis that the topical administration of insulin can drive RGC dendritic recovery and enhance RGC function in a murine model of glaucoma [36]. While the rectification of neuronal energy deficits presents an attractive therapeutic target this should be approached with caution. Our data confirm the importance of neuronal energy states in maintaining neuronal dendritic structure. It is possible that dendritic atrophy presents an adaptive response to injury so that axonal connections can be maintained [37]. In the zebra fish, for example, axonal regeneration is delayed by dendritic recovery, raising the possibility of a complex interplay between afferent and efferent neuronal connections (however, it is worth noting that the zebrafish is pro-neuroregenerative and that these mechanisms may not be the same in mature mammalian systems).

It is important to note that nicotinamide treatment at both doses was well tolerated by normotensive rats. This experimental paradigm demonstrates that even in the absence of a clear insult or nicotinamide deficient physiological state, nicotinamide supplementation does not exert any harmful effect [38]. On the contrary, we observed an increase in dendritic complexity suggesting that that retinal ganglion cell plasticity can be bolstered by nicotinamide supplementation. The consequence of increased plasticity in the absence of ocular hypertensive damage requires further investigation. However, since age-related RGC atrophy is normal physiological process which manifests with age-dependent visual deficits [39] it is reasonable to consider the use of nicotinamide in elderly patients to maintain retinal physiological function.

Metabolic profiling of the optic nerves confirmed that oral nicotinamide treatment substantially affected NAM metabolism in the optic nerve and that ocular hypertension itself had a marked effect on NAD metabolism. Further pathway analysis identified defective glycolysis and gluconeogenesis, supporting the rationale that bioenergetic decline can underpin neuronal degeneration in glaucoma. This is further supported by a recent Phase II trial in which glaucoma patients were treated with a combination of nicotinamide and pyruvate (the final product of glycolysis) which demonstrated an increase in visual function [40–42].

Our metabolomic data identified high NMN and low NAD + following ocular hypertension which was potently reversed by high dose oral nicotinamide treatment. SARM1, an important driver of axon degeneration [3] is allosterically inhibited by NAD and activated by NMN. When the NMN:NAD ratio favours NMN, SARM1 is activated and axon degeneration is initiated [43]. High NMN levels may be enough to drive neurodegeneration alone [44]. Since NAM treatment normalised the NMN:NAD in ocular hypertensive eyes it is reasonable to conclude that this would have a beneficial effect on axon survival. Even so, it is unlikely that neuroprotection is derived solely from SARM1 inhibition given that SARM1 KO in mice saves only retinal ganglion cell axons and not somas in glaucoma [45].

## Conclusions

Our data support the importance of nicotinamide metabolism and linked pathways as key components in retinal ganglion cell neuroprotection. The data confirm that dietary supplementation with nicotinamide provides dose dependent, robust protection of retinal ganglion cell structure following ocular hypertension and that this protection is complete, at the dendritic level, with high dose oral NAM.

### Supplementary Information

Supplementary Material 1

Supplementary Material 2

## Data Availability

All data generated or analysed during this study are included in this published article.

## Abbreviations

BDNF: Brain derived neurotrophic factor
CNS: Central nervous system
DiD: 1,1′-Dioctadecyl-3,3,3′,3′-tetramethylindodicarbocyanine, 4-chlorobenzenesulfonate
DiI: 1,1′-Dioctadecyl-3,3,3′,3′-tetramethylindocarbocyanine perchlorate
DiO: 3,3′-Dioctadecyloxacarbocyanine perchlorate
F6P: Fructose-6-phosphate
G6P: Glucose-6-phosphate
GSH: Glutathione
GSSG: Glutathione disulfide
HT: Hypertension
IOP: Intraocular pressure
KEGG: Kyoto encyclopaedia of genes and genome
MBI: Microbeads injection
NAD: Nicotinamide adenine dinucleotide
NADH: Reduced form of nicotinamide adenine dinucleotide
NAM: Nicotinamide adenine mononucleotide
NAMPT: Nicotinamide phosphoribosyl transferase
NMN: Nicotinamide mononucleotide
NMNAT: Nicotinamide mononucleotide adenine transferase
OHT: Ocular hypertension
PBS: Phosphate buffer saline
PCA: Principal component analysis
PFA: Paraformaldehyde
RCTs: Randomised control trials
RGC: Retinal ganglion cell
SARM1: Sterile alpha and Toll/interleukin-1 receptor motif-containing 1
